# Supplementary material for: Dissection of figured wood trait in curly birch (Betula pendula Roth var. carelica (Mercklin) Hämet-Ahti) using high-throughput genotyping
Source: Sci Rep. 2024 Mar 1;14:5058. doi: 10.1038/s41598-024-55404-y (PMC10904815; doi:10.1038/s41598-024-55404-y)
Supplement: Supplementary file 1 — Supplementary Legends. [file 41598_2024_55404_MOESM1_ESM.pdf]

Supplementary table S1 - List of samples, barcodes and phenotypes.
Supplementary table S2 – List of primers used for Sanger sequencing.
Supplementary table S3 - Genotype data for 37,045 SNPs across studied cohort (192 trees).
Supplementary table S4 - An annotated region associated with the curly phenotype.
Supplementary table S5 - SNP validation results.
Supplementary figure S6 - PCR analysis of full-sib progenies from Karelian birch crosses with the primers flanking SNP S10_3472479.
Supplementary figure S7 - PCR analysis of full-sib progenies from Karelian birch crosses with the primers flanking SNP S10_3465040.
