# Supplementary material for: Dissection of figured wood trait in curly birch (Betula pendula Roth var. carelica (Mercklin) Hämet-Ahti) using high-throughput genotyping
Source: Sci Rep. 2024 Mar 1;14:5058. doi: 10.1038/s41598-024-55404-y (PMC10904815; doi:10.1038/s41598-024-55404-y)
Supplement: Supplementary file 7 — Supplementary Figure S6. [file 41598_2024_55404_MOESM7_ESM.pdf]

PCR analysis of full-sib progenies from Karelian birch crosses with the primers flanking SNP S10_3472479
forward 5′- TCCTTCGGTCGATGAAATAACA and reverse 5′- CAATCTGCGAGAGGGAACAA
Phenotyped: cw – curly wood, ncw – non-curly wood

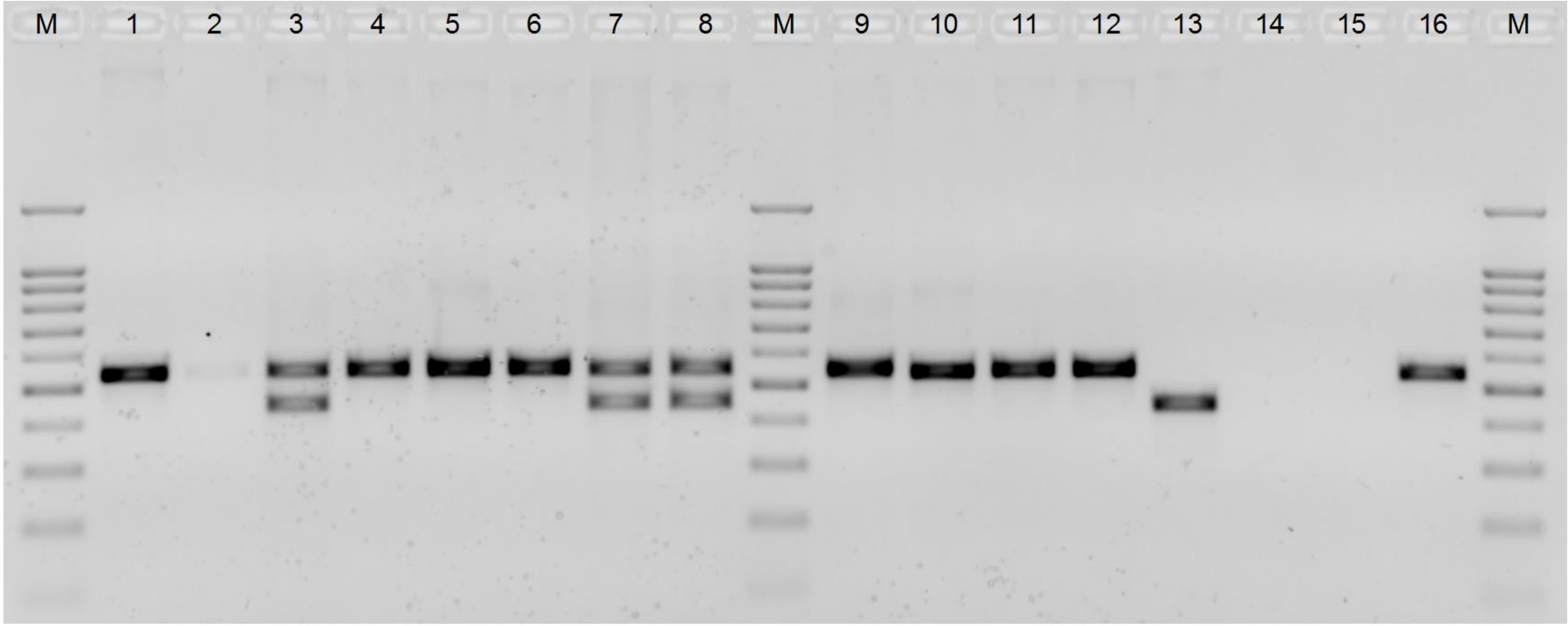

curly	cw	cw	cw	cw	cw	cw	cw	cw		cw	cw	cw	cw	ncw	cw	cw	cw

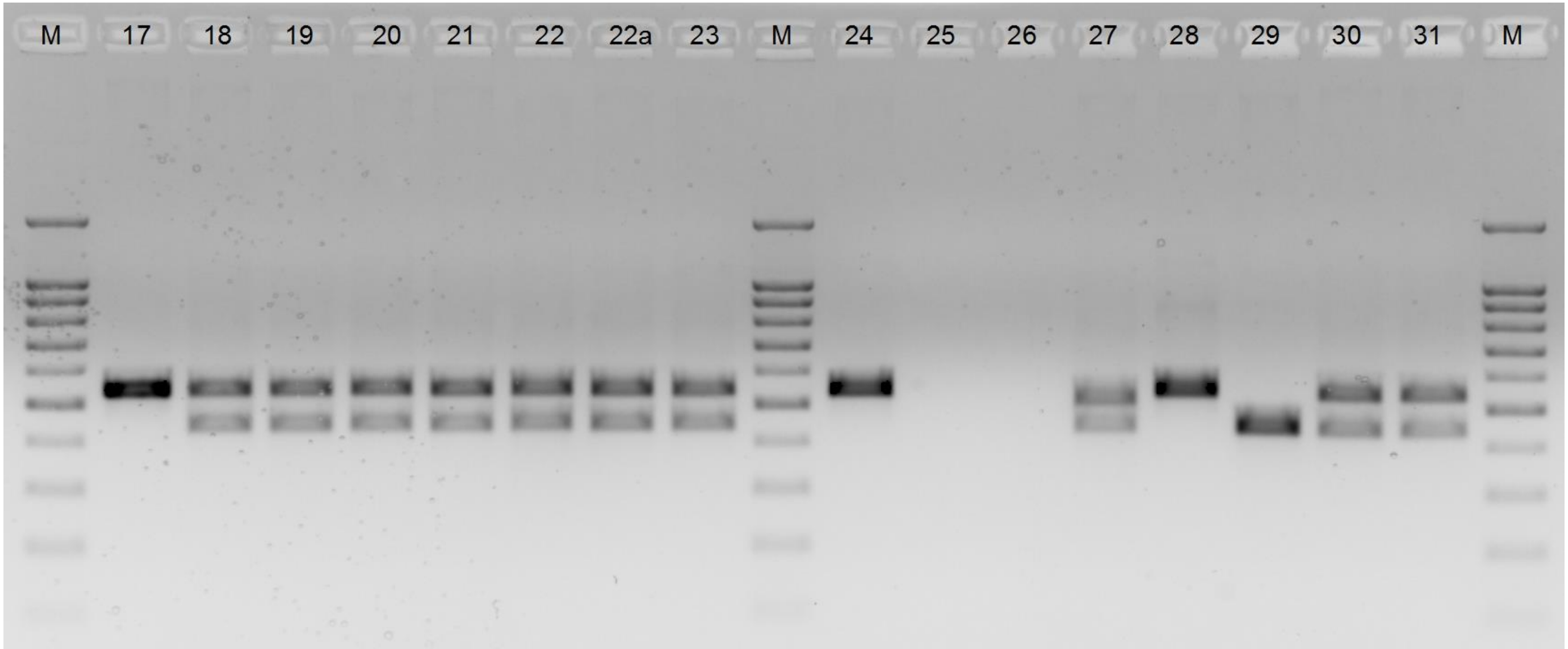

curly	cw	cw	cw	cw	cw	ncw	ncw	ncw		cw	cw	cw	ncw	cw	ncw	cw	cw

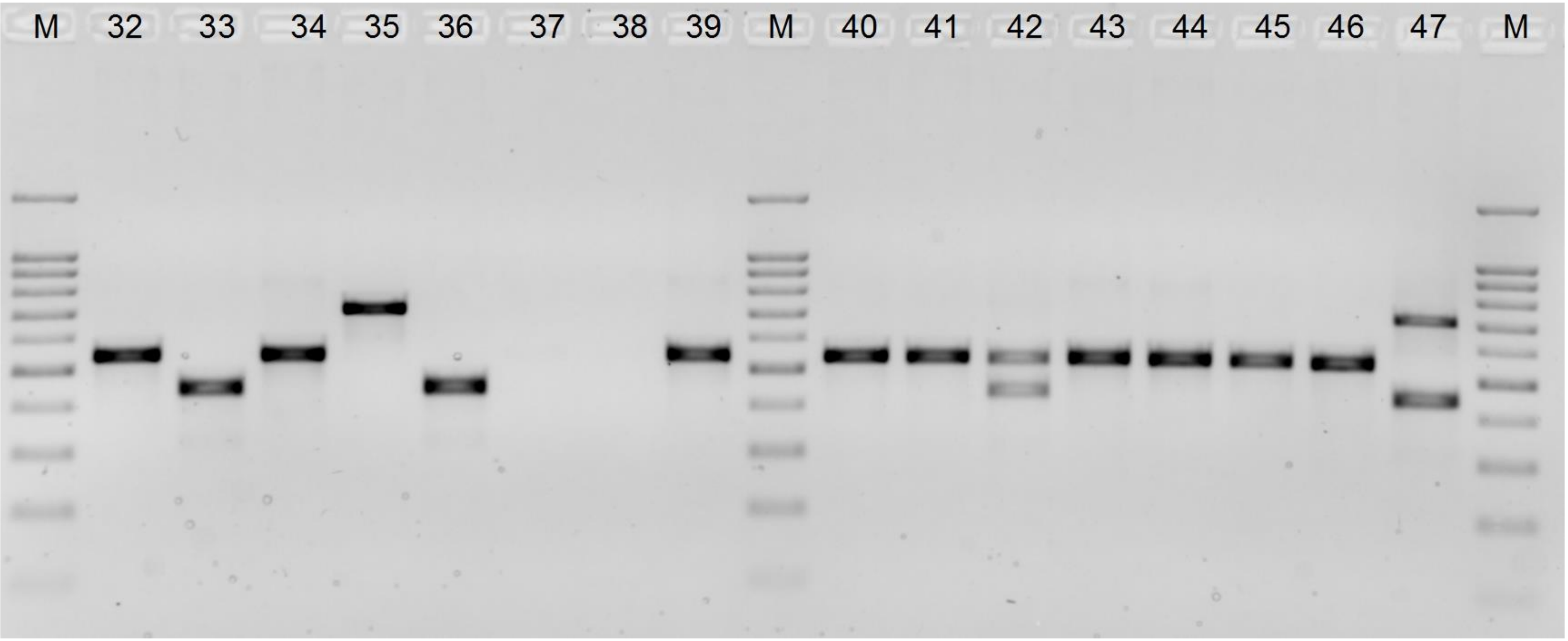

curly	cw	ncw	ncw	ncw	ncw	ncw	cw	cw		cw	cw	ncw	cw	cw	cw	cw	ncw

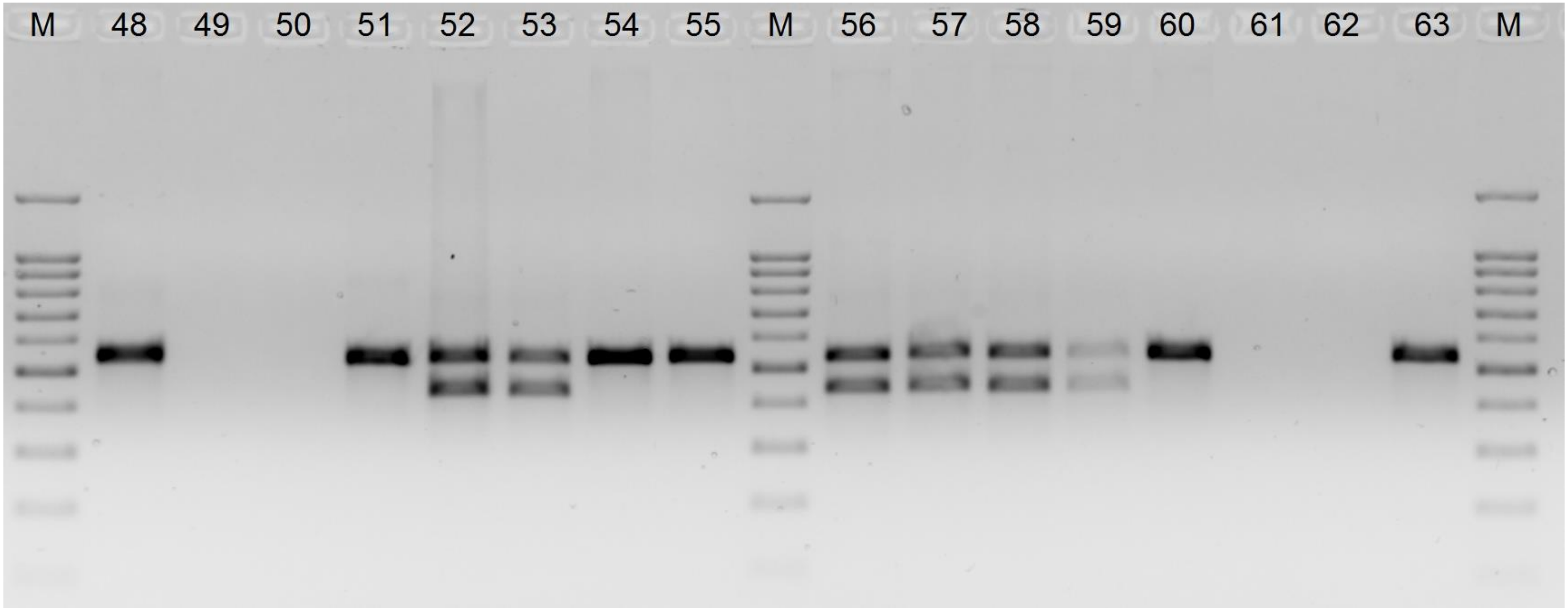

curly	cw	cw	cw	cw	cw	cw	cw	cw		ncw	ncw	cw	cw	cw	cw	cw	ncw

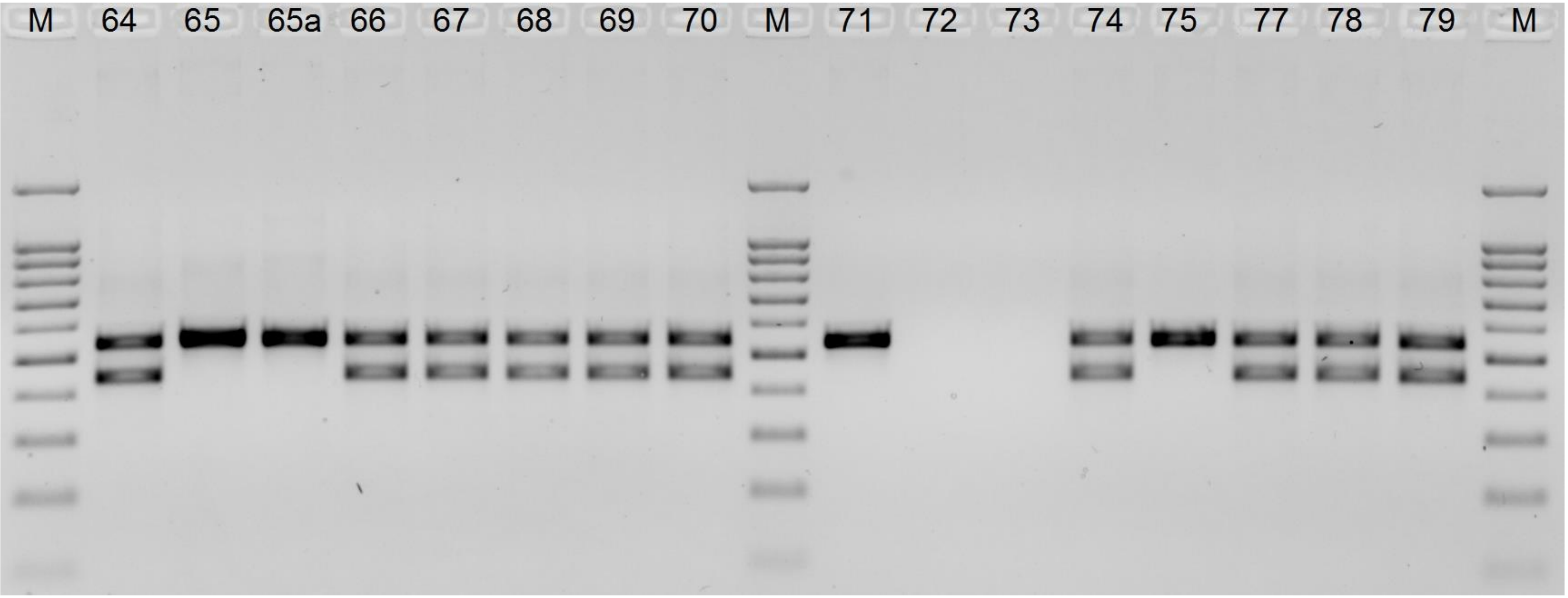

curly	ncw	cw	cw	cw	cw	cw	cw	cw		cw	cw	cw	cw	cw	cw	cw	cw

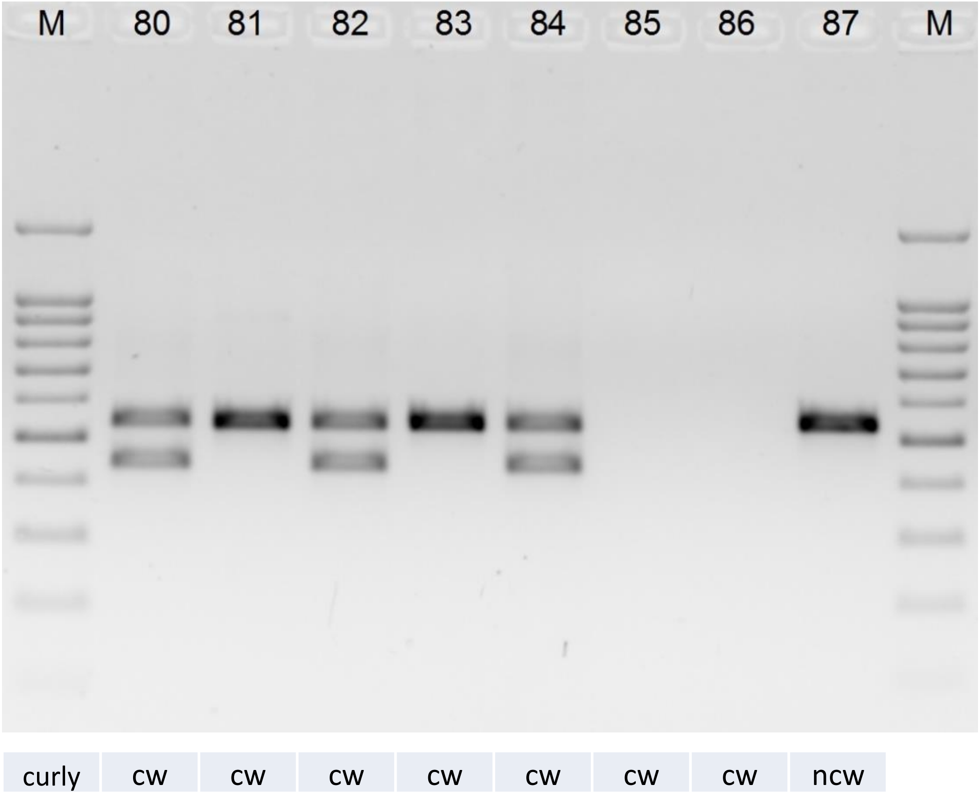
M 80 81 82 83 84 85 86 87 M
curly cw cw cw cw cw cw cw ncw

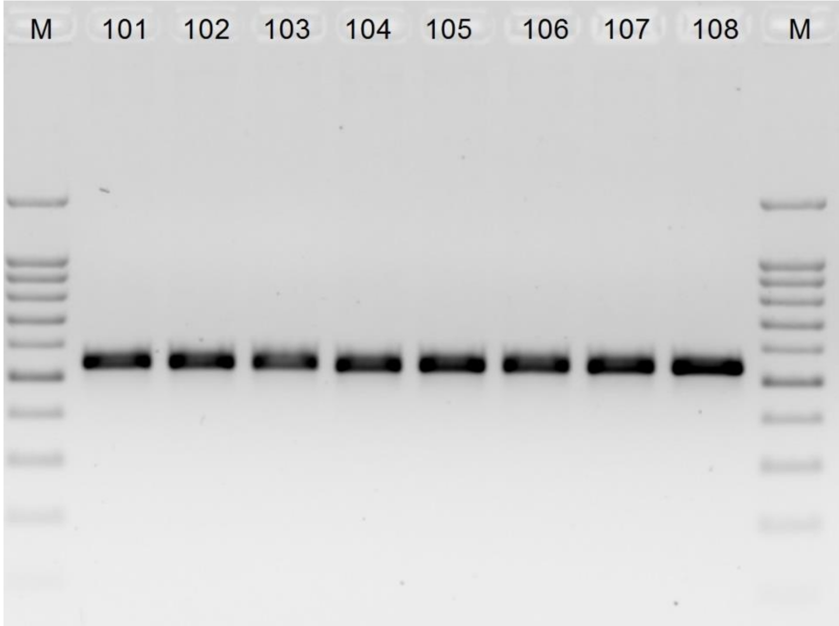

curly	cw	cw	cw	ncw	cw	ncw	cw	cw

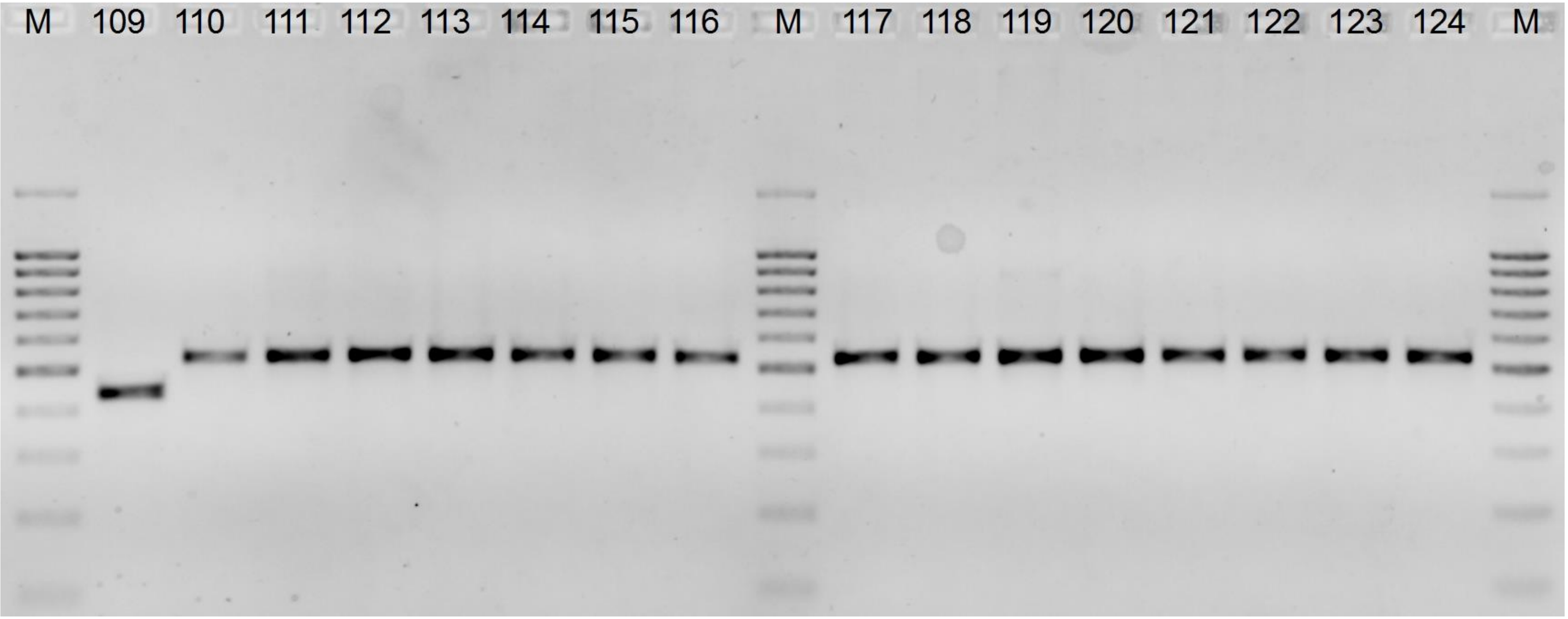

curly	ncw	ncw	cw	cw	cw	ncw	ncw	ncw		cw	cw	cw	cw	cw	ncw	ncw	ncw

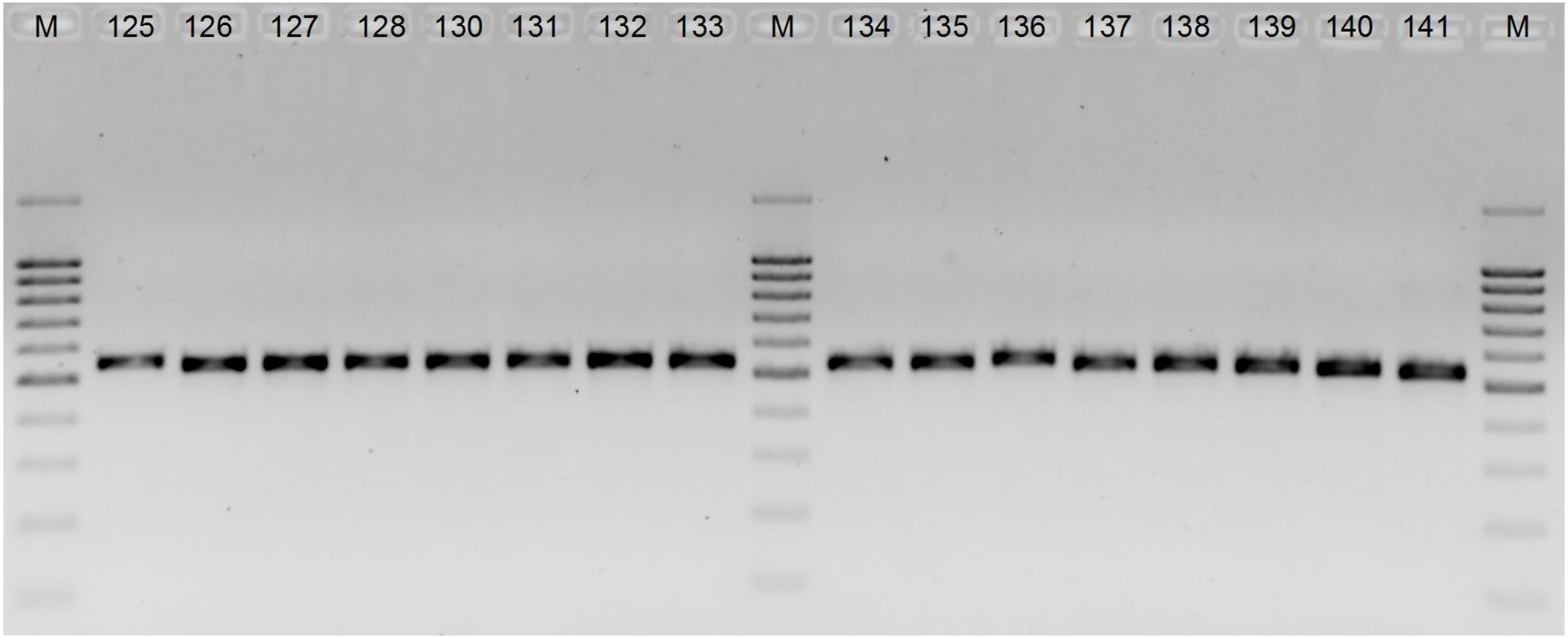

curly	ncw	ncw	cw	cw	cw	ncw	cw	ncw		ncw	ncw	ncw	cw	cw	cw	cw	cw

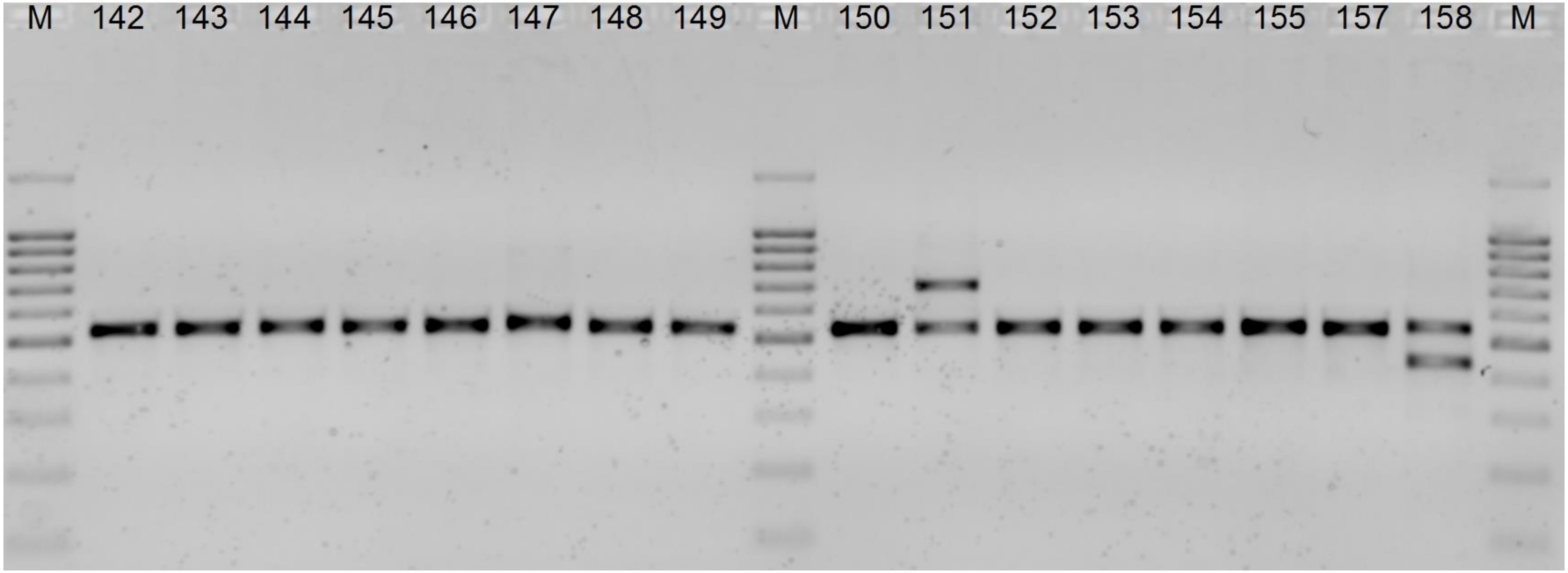

curly	ncw	ncw	ncw	ncw	cw	ncw	cw	cw		cw	cw	ncw	cw	ncw	ncw	ncw	ncw

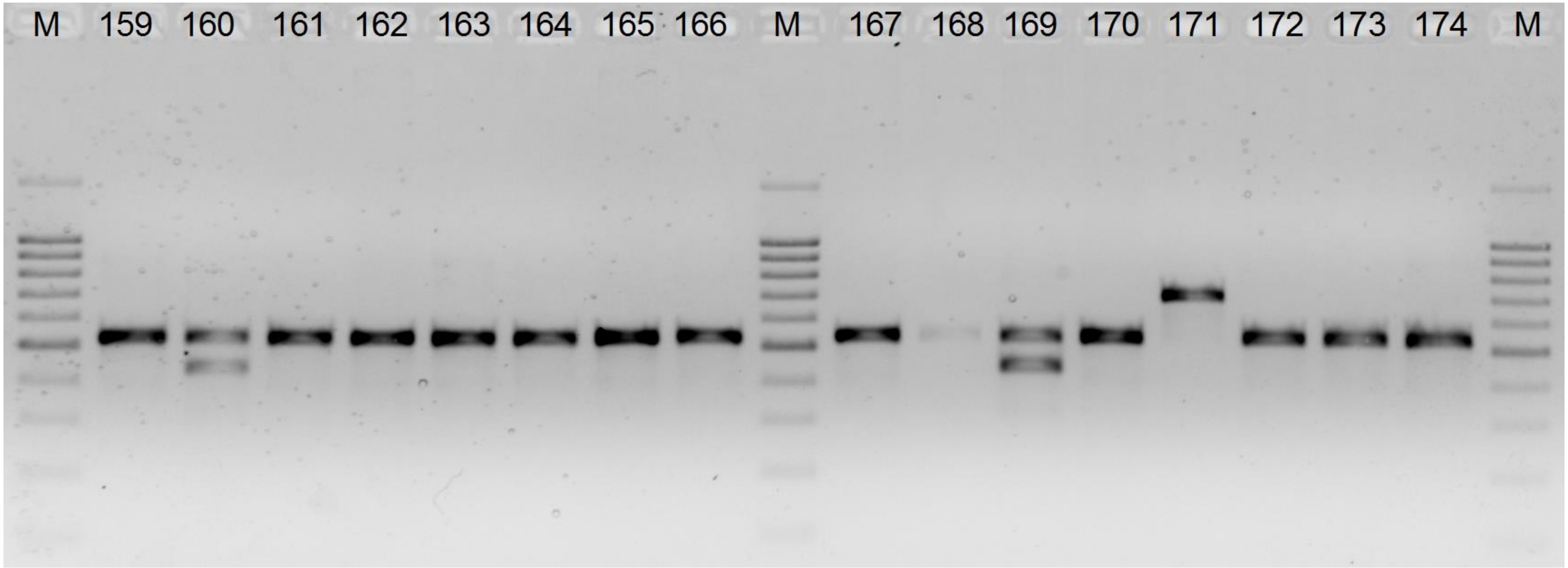

curly	cw	cw	cw	ncw	cw	cw	cw	ncw		cw	ncw	ncw	cw	ncw	cw	ncw	cw

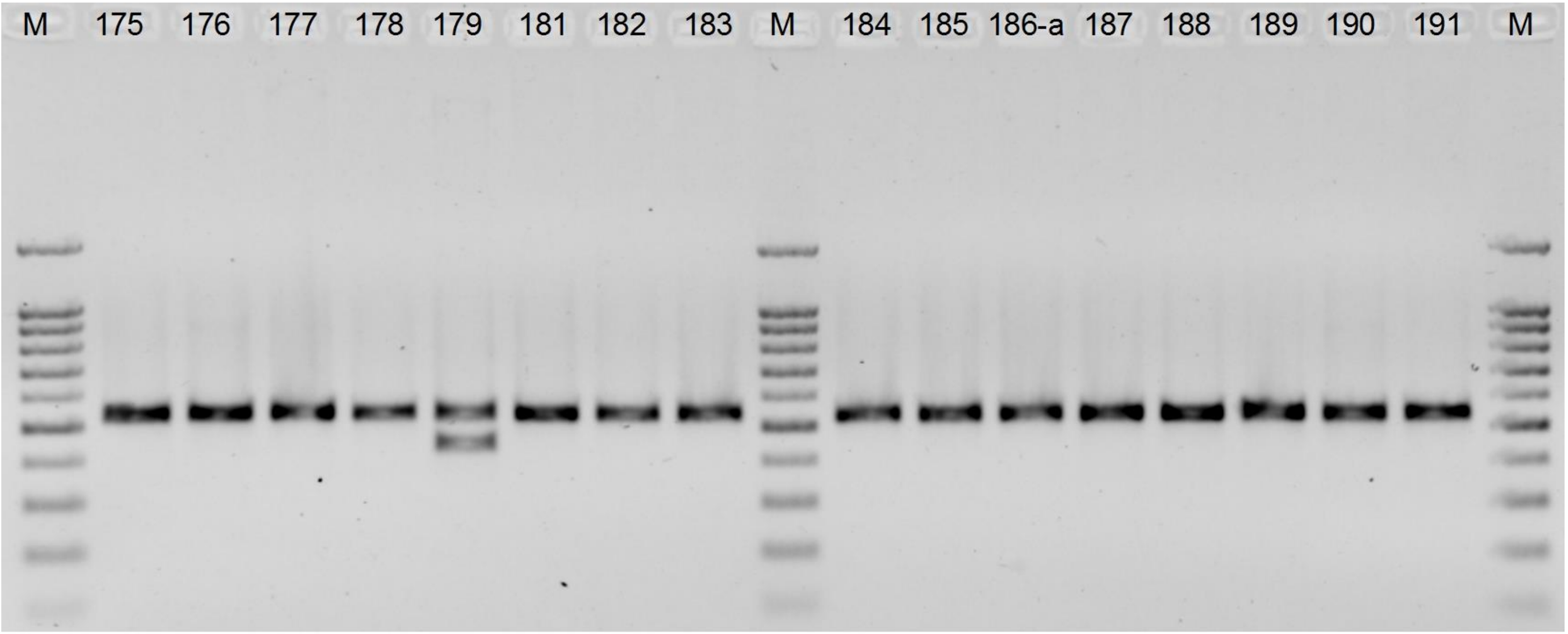

curly	ncw	cw	cw	ncw	ncw	cw	ncw	ncw		ncw	ncw	cw	cw	cw	cw	cw	ncw

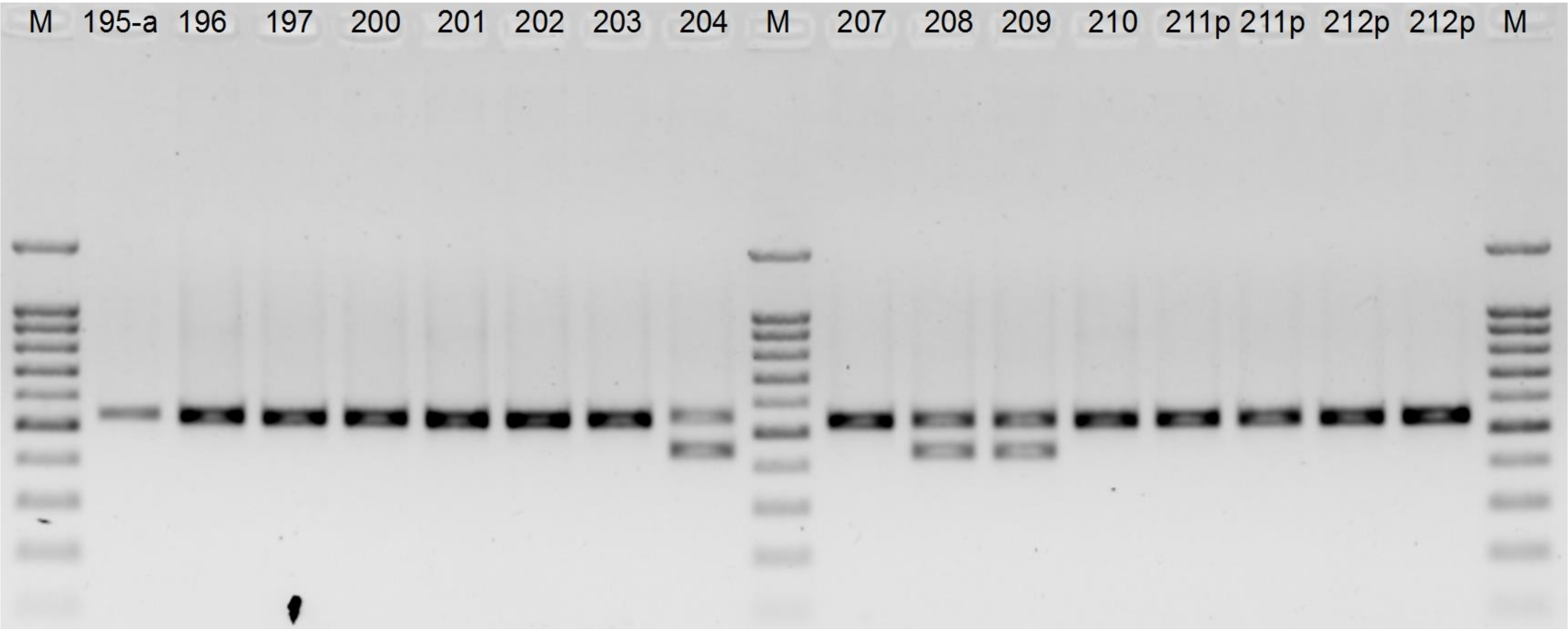

curly	ncw	cw	cw	cw	ncw	cw	cw	ncw		ncw	cw	cw	cw	cw	cw	cw	cw
